# Abnormal central venous oxygen saturation in cardiac surgery *patients:* a prospective, observational study

**DOI:** 10.1186/1749-8090-8-S1-O163

**Published:** 2013-09-11

**Authors:** D Unic-Stojanovic, P Vukovic, D Nezic, M Filipovic, P Milojevic, M Jovic

**Affiliations:** 1 Cardiovascular Institute Dedinje Belgrade, Serbia; 2 Medical faculty Belgrade, Serbia

## Background

Central (ScVO2) and mixed venous oxygen saturation (SvO2) and blood lactate are useful measurement tools for evaluating the degree of hypoperfusion in patients with different disease processes. The aim was to study incidence of both low as well as high ScvO2 and assessed their relationship to markers of tissue hypoxia, course and outcome in patients undergoing elective cardiac surgery.

## Materials and methods

Prospective, observational study. Settings: A 22-bed heart surgery intensive care unit (ICU) in a tertiary university hospital. Postoperative blood samples for the measurement of ScvO2 and lactate were obtained on arrival, 8 and 24 hours after ICU admission. 54 patients were divided into 3 groups based on ScvO2 values upon admission to the ICU: low, normal and high (‘‘supranormal’’) ScvO2 group. Data analysis was performed using the SPSS software package, version 20.0.

## Results and discussion

Total number of patients included in the study was 54 (41 males, 13 females), average age of 61.7±9.7 years old. Abnormal ScvO2 were recorded in 34/54 (62.9%) patients. A low ScvO2 (< 60.5) was recorded in 21 (38.8%) and high ScvO2 (>72.1) was recorded in 13 (24.1%) patients. While heart rates and central venous pressure were comparable between groups at all time points, mean arterial pressure was lower in the high ScvO2 group in comparison with the low and normal ScvO2 group. In the supranomal ScvO2 group there were higher lactate values, blood glucose levels, white blood cell count and the use of inotropes, so as prolonged ICU and hospital stay, compered to normal ScvO2 group.

## Conclusions

Supranormal values of ScvO2, which are traditionally considered to be of limited clinical value, turned out to be under-recognized warning signs of impaired tissue oxygenation in cardiac surgery patients. Further studies are necessary to assess the utility of ScvO2 and lactate to guide hemodynamic optimization and the impact it has on morbidity and mortality.

